# Effects of protein interaction data integration, representation and reliability on the use of network properties for drug target prediction

**DOI:** 10.1186/1471-2105-13-294

**Published:** 2012-11-12

**Authors:** Antonio Mora, Ian M Donaldson

**Affiliations:** 1 Department for Molecular Biosciences, University of Oslo, P.O. Box 1041, Oslo, Blindern 0316, Norway; 2 The Biotechnology Centre of Oslo, University of Oslo, P.O. Box 1125, Oslo, Blindern 0317, Norway

## Abstract

**Background:**

Previous studies have noted that drug targets appear to be associated with higher-degree or higher-centrality proteins in interaction networks. These studies explicitly or tacitly make choices of different source databases, data integration strategies, representation of proteins and complexes, and data reliability assumptions. Here we examined how the use of different data integration and representation techniques, or different notions of reliability, may affect the efficacy of degree and centrality as features in drug target prediction.

**Results:**

Fifty percent of drug targets have a degree of less than nine, and ninety-five percent have a degree of less than ninety. We found that drug targets are over-represented in higher degree bins – this relationship is only seen for the consolidated interactome and it is not dependent on n-ary interaction data or its representation. Degree acts as a weak predictive feature for drug-target status and using more reliable subsets of the data does not increase this performance. However, performance does increase if only cancer-related drug targets are considered. We also note that a protein’s membership in pathway records can act as a predictive feature that is better than degree and that high-centrality may be an indicator of a drug that is more likely to be withdrawn.

**Conclusions:**

These results show that protein interaction data integration and cleaning is an important consideration when incorporating network properties as predictive features for drug-target status. The provided scripts and data sets offer a starting point for further studies and cross-comparison of methods.

## Background

Drug targets (DTs) are defined here as proteins targeted by drugs. These proteins are not necessarily the products of disease-linked genes (which we will call Disease Proteins, DPs) but can be any protein whose binding might lead to a positive effect in the treatment of a disease. Yildirim *et al.* have presented a distinction between etiological and palliative drugs (the first targeting the DP or its neighbourhood, and the second attacking a different part of the network, probably to counteract symptoms of the disease-related proteins), and state that most known drugs are palliative [1]. This diversity of ways of treating a disease raises an important question: What are drug targets and why do they work? And can we predict them to help drug discovery?

Several studies have attempted to characterize drug targets from a theoretical point of view as such knowledge could be a tool to speed up the drug discovery process. Bioinformatics methods to characterize and predict drug targets have included: pathway and tissue enrichment, domain enrichment, number of exons and protein degree in an interaction network [2], GO enrichment [3], sequence similarity to known targets [4], side-effect similarity [5], physicochemical properties of the sequence of known drug targets [6], entropies of tissue expression and ratios of non-synonymous to synonymous SNPs [7], methods based on drug similarity, target similarity and network similarity [8,9], in addition to traditional text and data mining approaches [10]. These studies include network-based and non-network-based prediction methods, supervised and non-supervised, from those using the protein interaction space to those including chemical and pharmacological spaces, from single metrics to elaborated predictors with multiple features. Their predictive power has been evaluated by metrics such as the sensitivity, specificity or accuracy, and, specially, the Receiver Operating Characteristic (ROC), which has been widely used during recent years [6,11-13].

Drug targets can also be characterized in terms of protein network attributes such as degree and centrality. The degree of a protein in a protein interaction network is equivalent to the number of interactions a protein is involved in, while centrality measures quantify the relative importance of a protein. Types of centrality measures include Betweeness Centrality (according to the number of shortest paths that go through it) and Closeness Centrality (the shortest distance between that protein and all others). A number of studies have investigated drug targets in terms of such network based metrics including degree, betweenness centrality [7], bridging centrality [14] and pathway closeness centrality [15]. These studies reported significant differences between drug targets and non-drug targets suggesting that these network-based properties might be useful in predicting drug targets. For example, Zhu *et al.*[16] had some success using an assembly of network metrics (including degree) to train a support-vector machine to rank potential drug targets from the human proteome. This study used only those interactions contained in BioGrid to generate network metrics for proteins and they reported that 94 of their 200 top-ranked proteins were drug targets known to DrugBank.

The initial goal of this paper was to evaluate the predictive value of two simple graph-theoretical metrics, degree and centrality, that previously have been observed to correlate with drug targets [2,7,16-18] - the analysis could be extended to other network based prediction metrics. A number of observations have been made from these studies: drug targets are more likely to interact with more than 3 partners for FDA-approved drugs than non-approved [2], drug targets have high degree and centralities [17], drug targets have higher degree but far from the highest [7], drug targets have higher Betweenness Centrality [7], and more than 40% of drug targets are involved in 1 pathway [2]. In contrast, Hase *et al.*[18] claim that middle to low degree nodes happen to be advantageous targets.

These studies suggested network-based metrics might be useful for drug target prediction; however, the disparate conclusions (drug-targets are high-degree, middling degree or low-degree) was confusing. In trying to reproduce some studies, we commonly had difficulties determining exactly what data sets were used and found that studies often reported average drug target degree instead of entire degree *distributions* making it difficult to compare results between studies. We hypothesized that the distribution of graph-based metrics might be very dependent upon the choice of data. So the second goal of this paper was to use an exploratory data analysis approach to ask how network-based metric distributions changed when using various subsets of a well-defined, consolidated data set called iRefIndex [19]. The iRefIndex is a consolidated non-redundant dataset of 13 protein interaction databases (BIND [20], BioGrid [21], CORUM [22], DIP [23], HPRD [24], InnateDB [25], IntAct [26], MatrixDB [27], MINT [28], MPact [29], MPIDB [30], MPPI [31] and OPHID [32]), that examines the sequence of each protein in order to detect redundancies.

The studies above that have investigated network based metrics of drug targets rely upon PI data, and explicitly or tacitly make choices of different source databases, data integration strategies, representation of proteins and complexes, and data reliability assumptions. Previous work from our group [33] has shown the susceptibility of the graphical properties of a protein interaction network (PIN) to variables such as the number of included databases, redundant information between databases, canonical representation of proteins, complex representation, and reliability of included information, which makes this an important issue when comparing results from different drug target prediction studies.

Here, we examined the effect of data integration on the distribution of drug targets across degree and centrality measures (and the ability of these measures to predict drug targets). The above mentioned studies work with limited data sets: Yildirim *et al.*[1] use two high-throughput papers [34,35], which correspond to 8.2% of all known human interactions present in the consolidated interaction database iRefIndex [19], while both Sakharkar *et al.*[2] and Zhu *et al.*[16] use the BioGrid database [21], which corresponds to 15.7% of human interactions in iRefIndex, and Hase *et al.*[18] use results from one study [34], which correspond to 3.8%. We hypothesized that different conclusions might be reached just by using the complete iRefIndex data set.

Next, we examined the effect of sub-setting interaction data upon the drug target distribution over proteins of varying degree and centrality. We hypothesized that using subsets of interaction data deemed to be more reliable might alter this distribution and be useful for the purpose of predicting drug targets. There are several methods used to rank protein interactions according to some specific notion of reliability. Early attempts include the Expression Profile Reliability (EPR index), which compares protein interaction and RNA expression profiles, and the Paralogous Verification Method (PVM) that searches after paralogs of interactors which also interact (Deane *et al.*[36]). In this paper, we examined five methods that have been argued to change the reliability profile of data. The first method is a bibiliometric-based measure called “lpr” [19,33,37,38] which is able to distinguish high-throughput and low-throughput experiments. It has been suggested that low-throughput studies contain a higher rate of reliable interactions than high-throughput studies [39] although this conclusion has been contested [40]. The second and third methods are two annotation-based scores generated by Intact and by multiple PSICQUIC services [41], which take into account the number of publications supporting an interaction, number and type of experimental methods, and interaction types [42]. As a fourth method, we considered the effect of removing all n-ary derived interactions from our data set. N-ary (aka complex) interactions are created by a family of interaction-detection methods that show that a set of proteins are somehow interacting without specifying the exact binary interactions involved. N-ary derived interactions include binary interaction records that are actually spoke or matrix representations of n-ary data and we have shown that the inclusion of such data can alter graphical properties of a network [33]. Finally, we considered the effect of removing all predicted interactions (by orthologous transfer) from our consolidated data set - iRefIndex includes the predicted interaction database OPHID [32]. Each of these five “more reliable” datasets was examined in terms of their effects on the distribution of drug targets across bins of proteins of varying degree and centrality. Further, each distribution was assessed in terms of its effect on degree and centrality as drug target predictors.

Further, we addressed the effect of representing all n-ary data using a spoke-model representation (where only interactions between each member of the group and one chosen protein are included) versus a matrix-representation (where all possible pair-wise interactions between the group of proteins are included [33,38]). The representation of n-ary data is not always apparent in a study, but we know that this choice has consequences for network properties [33].

Finally, we consider the drug target predictive ability of pathway data – a data source that is overlapping but complementary to interaction data. This partial overlap drew our attention to the usefulness of pathway data to drug target prediction, and motivated us to consider a pathway-degree metric for proteins.

In summary, we have chosen degree and centrality as simple drug target predictor features, in order to study the validity of the conclusions about them found in the literature when we work with consolidated protein interaction data from iRefIndex and various decisions regarding data integration, representation and reliability. We have previously shown that network properties can be altered by these choices and we will show the potential effect of these factors on drug target prediction.

## Results

Our results section is divided into five parts which examine: 3.1) integration, 3.2) selection, 3.3) representation, 3.4) pathway data and 3.5) relationship to diseases. In order to compare the effect of the source of data on the results, a series of human PINs were generated from the iRefIndex database [19] using the iRefR package [33], as specified in the Methods section. R code used to perform each analysis and to create each table and figure in the paper is provided in Additional file 1.

### Data integration

Here we test two hypotheses: First, that the high degree observed in drug targets might be related to the fact that specific databases or papers were chosen instead of a consolidated database and, therefore, this correlation might disappear after data integration, i.e., when using the iRefIndex. Secondly, that the high degree of some drug targets could be related to the inclusion of n-ary data.

#### Drug targets are correlated to high-degree only in the full data set

In order to evaluate if drug targets are on average high degree proteins in a consolidated PIN, we compared the average degree of all nodes to drug targets in the full PIN. Table 1 shows that the average degree of *just* drug targets (22.5) is higher than that of all nodes in the full network (14.2), while *just* non-drug targets are similar to the full case (13.5). The skewness and kurtosis values show that the full PIN has a peaked and right-skewed degree distribution with the drug targets having a more peaked and skewed distribution than non-drug targets. The most-highly connected protein of the full network is itself a drug target with degree 789 (Grb2 protein), and only 23 of these interactions are with other drug targets.

**Table 1:** Degree of all proteins, drug targets only and non-drug targets only for the full PIN and various subsets

**Protein interaction network**	**Nodes examined**	**Mean degree**	**Median degree**	**Degree standard deviation**	**Degree skewness**	**Degree kurtosis**	**Max degree**
full PIN -spoke	all	14.2	4	28.9	6.6	86.1	789
	drug targets in full PIN	22.5	8	44.8	6.8	84.2	789
	non-drug targets in full PIN	13.5	4	27.1	6.0	66.5	615
drug target subnetwork -spoke		1.7	1	44.7	3.2	15.8	23
non-drug target subnetwork -spoke		12.7	4	26.3	6.8	90.3	709
BioGRID only –spoke	all	7.5	3	13.5	7.9	133.0	395
	drug targets in BioGRID only	9.0	3	18.6	5.5	41.3	203
Rual + Stelzl papers only -spoke	all	4.3	2	8.8	7.5	81.6	158
	drug targets in Rual + Stelzl only	3.7	2	5.5	6.0	54.7	60
Rual paper only –spoke	all	3.8	2	8.4	9.4	127.5	158
	drug targets in Rual only	2.2	1	2.5	3.5	19.4	15

Statistical descriptors of the degree distribution of 11 different PINs whose protein complexes have all been represented as spoke models (i.e. any N-ary data is included by a spoke-model representation). Drug targets have a higher degree on average, even though the standard deviations are equally higher. Degree distribution of drug targets are also more skewed and peaked than non-drug targets. This is different in distributions like the BioGRID database or the Rual and Stelzl papers, where the numerical values are not only significantly smaller but the conclusions might be even the contrary, such as drug targets having a lower degree for Rual and Stelzl. The values of the drug target subnetwork show that interactions between drug targets are scarce and, therefore, the average higher degree of drug targets represent interactions between drug targets and non-drug targets. BioGRID was used by [2], Rual+Stelzl was used by [1] and Rual-only was used by [18].

Given this observation, we examined the sub-graph consisting only of interactions between drug targets versus the non-drug target sub-graph. The average degree of the drug target sub-network is only 1.7 (versus 12.7 for the non-drug target sub-network), indicating that drug targets are, on average, high degree proteins more connected to other sites of the full network than among themselves.

For comparison purposes, the last six rows of Table 1 include the data sets of BioGRID, Rual and Stelzl, and only Rual, employed in other drug target studies [1,2,18]. It is evident that mean values are much higher for the full data-set than for any of these specific database or study subsets. Moreover, in the comparatively small Rual and Stelzl dataset, drug targets actually have an average degree that is lower than non-drug targets. In addition, skewness and kurtosis values indicate these smaller datasets are even more skewed than the full network case.

These initial results were consistent with drug targets having a higher degree on average in the consolidated dataset; however, the large standard deviation in these values led us to examine the relationship in greater detail. The majority of DTs have degrees between 1 and 8 (50th percentile) and 95% of all DTs have a degree less than 89. The number of DTs decrease linearly with degree between 1 and 20 followed by a long tail out to degree 789 (Additional file 2: Figure S1). A frequency plot shows that DT’s appear to be shifted to higher degrees compared to non-DTs and that this difference is significant (Wilcoxon p-value 6.5e-41) (Additional file 2: Figure S2).

**Figure 1:**
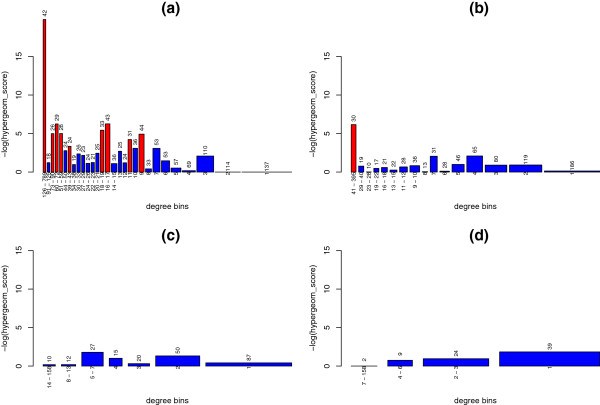
**Over-representation of drug targets over a degree ranking of proteins.** Proteins were grouped into bins according to their degree. The width of each bin represents the number of proteins in that bin while the height (−log of the p-value of the hypergeometric test) represents how over-represented drug targets are in that bin. Each bin contains at least 200 proteins. Over-represented bins (p-value < 0.05) are highlighted in red. The number of drug targets in each bin is indicated at the top of each bar. Drug targets are over-represented in high-degree bins and some middle-degree bins for the full PIN (**a**), while over-representation is observed only in the highest degree bin of BioGrid (**b**) and not at all in the Rual and Stelzl (**c**) or Rual-only (**d**) data sets.

In order to examine this overrepresentation in more detail, we constructed a rank of protein degrees in the full network and grouped them into bins of not less than 200 proteins each. Rank position 1 has the maximum degree of 789 and position 16078 has a degree of 1. We counted the number of drug targets per bin in the resulting 30 bins and applied a hyper-geometric test to each with a significant p-value cut-off of < 0.05. Figure 1 shows that bins where drug targets are over-represented (red) are mainly higher degree bins (left end of Figure). Numbers above each bar indicate the actual number of DTs in that bin; again while the number of DT’s in these higher-degree bins is quite low, their numbers appear to be overrepresented.

However, this trend is not seen in either the BioGrid or Rual and Stelzl sub-sets. In fact, drug targets were not over-represented at all in these two subsets with the exception of the highest degree bin in BioGrid. These observations argue that using degree as a feature for drug target prediction is significantly affected by choice of data-set.

The process of sub-setting the network will fragment it into smaller components containing drug targets that are disconnected from the main giant component. The full spoke human PIN contains 140 connected components, distributed as shown in (Additional file 3: Table S1), with one giant component including 15754 proteins. The giant connected component contains 1220 drug targets while all the others contain 7 drug targets altogether. A GO term analysis, using GO [43], revealed that proteins in these separate, small connected components are mainly located in the extracellular region and in the membrane with few in the cytoplasm or nucleus. Curiously, the drug targets in these smaller connected components are mainly cytoplasmic proteins. Consistent with this, the proteins in these disconnected components are mainly involved in cell adhesion, while the drug targets here are mainly involved in signal transduction. This suggests that they are not really independent functional modules but data with missing connections to the main connected component.

The connected component analysis in the different networks under study will show below how disconnected the network becomes when selecting reliable interactions. For example, the number of drug targets present in these smaller, disconnected components can go from 7 in the full PIN to 41 in the PSICQUIC MI-score subset (Table 2). As a result, sub-setting the data may remove a limited number of drug targets into disconnected components away from the main network.

**Table 2:** Number of drug targets present in isolated components in the full and reliable subsets

**Network**	**# Connected components**	**# Proteins in disconnected components**	**# Drug targets in disconnected components**
Full PIN	140	324	7
B subset	139	354	12
Non-predicted subset	164	376	12
MI score - IntAct	75	207	18
LTP subset	188	428	27
MI score - PSICQUIC	138	372	41

The full PIN contains 7 drug targets that are disconnected from the main component. This number increases as interactions are removed to generate subsets of the data that are potentially more reliable; i.e., more drug targets become disconnected from the largest component of the network.

#### Drug target degree is not overly influenced by n-ary data

We considered the possibility that the higher-degree of drug targets might be influenced by the presence of n-ary data in the full data-set. In a previous work, we distinguished between true binary data (B), n-ary also known as complex data (N) and spoke-represented n-ary data (S) [33]. The S type of data was defined as data records that are binary (only two interactors in the record), but that are in fact a spoke representation of n-ary data. Both N and S-type data could artificially inflate the degree of some nodes. Therefore, we separated these three data-types in the full network into three networks called B, N and S (see Methods) and re-examined the degree distribution of drug targets in each in order to rule out the possibility that the high-degree of drug targets is only due to the presence of n-ary type data.

**Figure 2:**
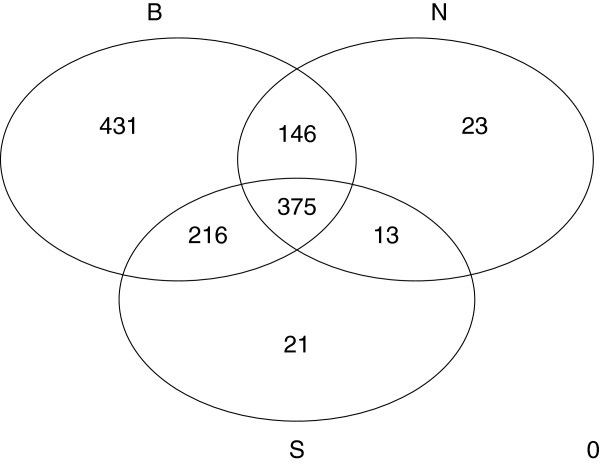
**Number of drug targets in each interaction-type subset. **Venn diagram with the number of drug targets per interaction type in the full spoke PIN. B corresponds to binary interactions, N to n-ary interactions and S to spoke-represented n-ary interactions. 431 drug targets are found only in the binary subset while 375 are found in all three subsets.

Figure 2 shows that only 57 out of 1225 drug targets belong to the non-B subset and, therefore, might be sensitive to complex representation. As a result, a network composed solely of binary interaction data covers almost the same number of drug targets as the whole network and a correlation between high degree and drug targets is not likely to be an artefact of complex representation but a property of real binary interaction data. Table 3 shows that all subsets are similarly enriched in drug targets (from 7.6% to 9%) but the largest share of drug targets is in the B subset (95%). The higher average degree of the B subset can be taken as an indicator that higher-degree proteins are also mainly located in the B subset while the N subset, the one subject to the complex representation problem, includes lower-degree proteins. The localization of both drug targets and high-degree proteins in the B subset can be taken as an indicator of a relationship between these variables and rules out the idea that this is only a protein complex representation issue.

**Table 3:** Drug target content and degree properties of the full network versus interaction type subsets

**Network**	**% All drug targets (#drug targets in data set / Total #drug targets)**	**% Drug targets in data set (#drug targets in data set / #proteins in data set)**	**Average degree of data set**	**Maximum degree**
B subset	95.19	8.11	10.44	534
N subset	45.39	8.98	7.37	282
S subset	50.94	8.78	7.57	169
Full PIN	100	7.63	14.16	789

Most drug targets are present in the binary (B) subset, while the n-ary (N) and spoke-represented n-ary data (S) subsets have around half of them. This might simply be due to the size of each subset, given that the ratio of drug targets to proteins per subset is similar. The average degree of the B subset is higher in comparison to the values for the N and S subsets, suggesting that the B subset may be a candidate to display a correlation between drug targets and high protein degree.

**Figure 3:**
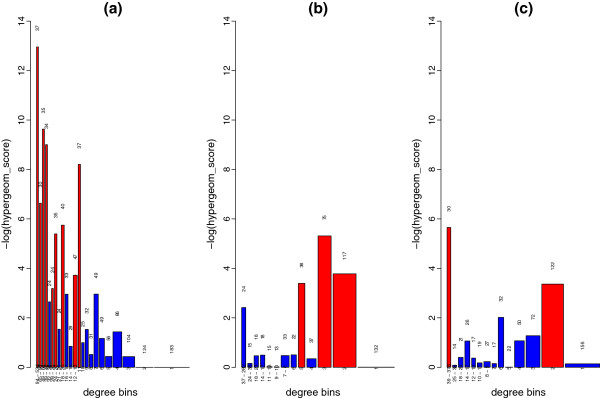
**Over-representation of drug targets along a degree rank for Binary (B), N-ary (N) and Spoke-represented (S) interaction types. **Proteins were grouped into bins according to their degree. The width of each bin represents the number of proteins in that bin while the height (−log of the p-value of the hypergeometric test) represents how over-represented drug targets are in that bin. Over-represented bins (p-value < 0.05) are highlighted in red. The number of drug targets in each bin is indicated at the top of each bar. Each bin contains at least 200 proteins. Drug targets are over-represented in high-degree bins and some middle-degree bins for the B subset (**a**), while this trend is largely lost in the N (**b**) and S (**c**) data sets.

The over-representation plot for drug targets in a degree rank for the B, N and S data sets confirms this. Figure 3 shows that the B data set displays drug target over-representation (red) in high-degree bins (left), in much the same way as the full PIN. However, such a trend cannot be seen in the N or S data sets. We conclude that n-ary data is not necessary to demonstrate the over-representation of drug targets in high-degree nodes.

#### Drug target association with higher centrality is dependent on n-ary data

We repeated the above analyses using betweenness centrality instead of degree (Table 4). Drug targets are, on average, proteins with high centrality values. Again this is dependent upon data-integration since the same is not apparent in either the BioGrid or Rual and Stelzl subsets. The average centrality of all nodes in the N subset of the data (records with 3 or more interactors) is higher than the full network since n-ary data was represented in a graph using a spoke model where one protein in each record is chosen as a hub to which all other members of the record are adjacent. The centrality measure for the n-ary data is very similar to that of just the drug targets in the full network (Table 4: compare line 2 with 5 and 6). This led us to believe that centralities in the full network might be more susceptible to inflation by the N and S subsets than in the case of the degree analysis.

**Table 4:** Betweenness Centrality properties for different human PINs

**Protein interaction network**	**Nodes examined**	**Average BC (per protein)**	**Maximum BC**
full PIN -spoke	all	21663.7	6930614.5
	Drug targets only	47319.8	6930614.5
	Non-drug targets only	19545.3	5195198.1
	B nodes only	23985.2	6930614.5
	N nodes only	46165.3	6930614.5
	S nodes only	43327.2	6930614.5
BioGRID subnetwork	all	13704.3	4436940.8
Rual+Stelzl subnetwork	all	5960.5	506957.4

BC behaves similar to degree in the sense that drug targets have higher centralities than non-drug targets, and in the sense that BioGRID and Rual-Stelzl display smaller values in comparison with the consolidated data set. However, a difference appears regarding interaction type, where nodes belonging to n-ary interactions (N and S nodes) are more central than nodes belonging to binary interactions.

We examined the distribution of drug target centralities (Additional file 2: Figure S3) and found that drug targets were indeed over-represented in higher centrality bins. However, and in contrast to the degree analysis, this trend was diminished in the absence of the N and S subsets. In contrast, these trends were largely absent from the N or S subsets themselves (i.e., binary data is required to see the drug target centrality trend) and from the two smaller subsets.

In summary, DT’s appear to be overrepresented in higher-degree and centrality bins. However this is most apparent using a consolidated data set and is somewhat dependent on the presence of n-ary data in the case of centrality. Most drug targets seem to be located in true binary interaction data and their degree distributions are therefore not likely to be affected by complex representation artefacts.

### Data selection analysis

We wished to quantify the predictive power of high degree and centrality for drug targets and assessed this using the Receiver Operating Characteristic (ROC) on the full-network. We then compared this performance over five different subsets of the data that could reasonably have an effect on reliability and on network properties with respect to the full network. Our rationale here was that removing unreliable data might decrease the degree for some non-drug targets that had been artificially inflated and thereby increase performance by removing false-positives.

**Figure 4:**
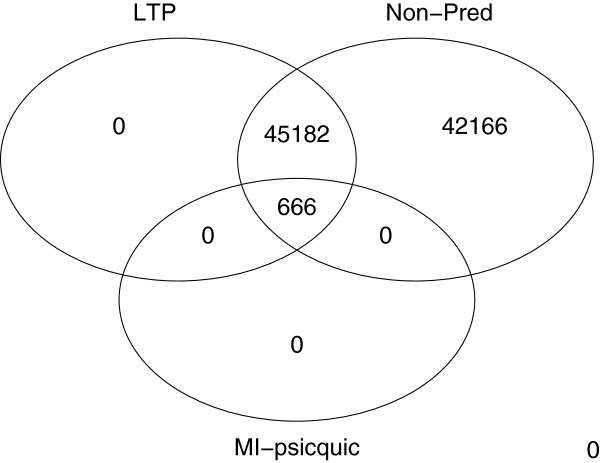
**Venn diagram of interactions found in three of the reliable subsets. **The Venn diagram shows that all MI-PSICQUIC interactions (MI-PSICQUIC score > 0.8) are contained in the LTP data set (lpr < 22), which in turn is contained in the non-predicted data set (data set excluding the OPHID database).

The “more-reliable” data sets included binary data only (B), data excluding predicted interactions (NP), low-throughput data only with an lpr < 22 (LTP), just edges with an MI score (IntAct) > 0.6 (I) and just edges with an MI score (PSICQUIC) of > 0.7 (P). The construction of each subset is described in the Methods section. Figure 4 shows that these subsets are not completely independent and that the different reliability measures may be detecting similar types of interactions: the subset of MI-PSICQUIC interactions is a subset of the LTP interactions, which in turn is a subset of the non-predicted interactions.

### Degree as a drug target predictor

**Figure 5:**
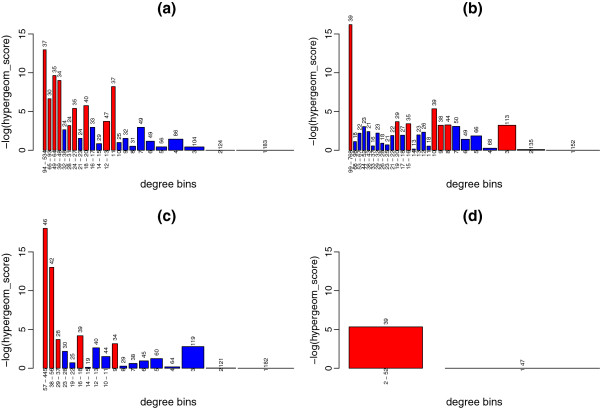
**Over-representation of drug targets along a degree rank for the Binary (B), non-predicted interactions, lpr < 22, MI-IntAct > 0.6 and MI-PSICQUIC > 0.8 data sets. **Proteins were grouped into bins according to their degree. The width of each bin represents the number of proteins in that bin while the height (−log of the p-value of the hypergeometric test) represents how over-represented drug targets are in that bin. Each bin contains at least 200 proteins. Over-represented bins (p-value < 0.05) are highlighted in red. The number of drug targets in each bin is indicated at the top of each bar. Drug targets are over-represented in high-degree bins and some middle-degree bins for the binary-only (B) subset (**a**), the non-predicted subset (**b**) and the LTP (lpr < 22) data set (**c**), while there is no over-representation for the MI-IntAct (MI-IntAct score > 0.6) (**d**) and the MI-PSICQUIC (MI-PSICQUIC score > 0.8) (e) data sets.

An over-representation plot of the five different data sets (Figure 5) shows that the B and LTP data sets still display over-representation of drug targets in high-degree bins while these trends are less clear for the non-predicted data set (NP). The low number of nodes in the high-scoring data (P and I data sets) leads to lower-degree distributions and does not allow for a conclusion or direct comparison.

**Figure 6:**
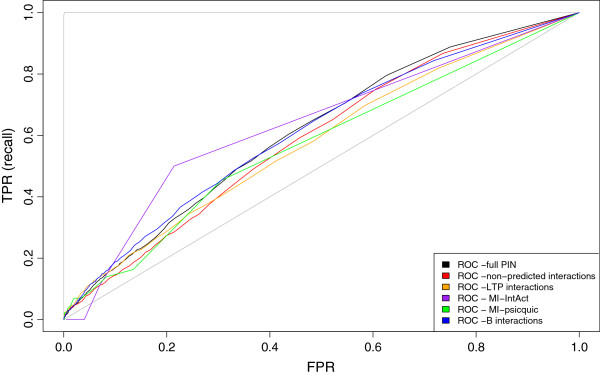
**ROC curve for protein degree as a drug target predictor. **Plot of False Positive Rate versus True Positive Rate for a degree rank of the full PIN and five subsets considered as containing higher-confidence interactions: non-predicted interactions include all interactions except those coming from orthologous transfer; LTP includes interactions with an lpr score < 22; MI-IntAct includes interactions with MI-IntAct scores > 0.6; MI-PSICQUIC includes interactions with MI-PSICQUIC scores > 0.7; and B includes the true binary interactions (i.e., potential spoke-represented n-ary data is removed). Theoretically perfect and random classifiers are shown in grey for reference (AUC = 1 and 0.5 respectively).

In order to quantify the predictive power of the degree for these data sets, we plotted the ROC curve (Methods) shown in Figure 6. Here, the perfect predictor would have an area under the curve (AUC) of 1, while the random case would be close to 0.5. The values of the AUC for the full PIN and the reliable subsets are shown in Table 5. Degree in the full network is indeed a predictor of drug targets, even though a modest one. The full network provides the best performance with one exception: the IntAct high reliability subset (I) scores slightly higher. However, this subset recovers only a small subset of drug targets. Due to this, we concluded that the effect of removing less-reliable interactions is not positive and prediction might work better with all interactions with a variety of degrees. In support of this, arbitrary subsets, like BioGRID or the Rual plus Stelzl papers, have a very poor performance, close to randomness.

**Table 5:** Drug target predictive power of degree and centralities for different reliable subsets

**Network**	**Number of proteins in network**	**AUC – Degree**	**AUC - BC**	**AUC - CC**
Full PIN, spoke	16078	0.6139	0.6294	0.5795
B subset	14408	0.6114	0.6171	0.5764
Non-predicted interactions	14928	0.5916	0.6128	0.5647
LTP subset	10591	0.5794	0.6066	0.5482
BioGRID only	8642	0.5082	0.5467	0.4874
MI score, IntAct > 0.6	219	0.6353	0.5347	0.4382
MI score, PSICQUIC > 0.7	747	0.5719	0.5725	0.5414
Rual+Stelzl only	3575	0.5004	0.5045	0.5011

The AUC was evaluated for degree and centrality ranks of the full PIN, five reliable subsets and two small subsets used in the literature. The best degree performance is achieved by the MI-IntAct score greater than 0.6; however, this subset contains 219 proteins only, making it of limited applicability. The second best performance is achieved by the full PIN and the B subset. Other reliable subsets (non-predicted, PSICQUIC, LTP) have a slightly inferior performance, while BioGRID and Rual+Stelzl perform close to randomness.

The best centrality performance is achieved by the full PIN, followed by three reliable subsets (B, non-predicted and LTP). Both MI-scores and both limited data sets perform close to randomness.

### Centrality Analysis

Over-representation of drug targets along a centrality rank for the full PIN and each of the subsets behave similarly to degree. We assessed Betweenness Centrality performance using AUC as described above and found results similar to the degree performance (Table 5). The full data set gave the best performance (AUC 0.63). A second measure of centrality (Closeness Centrality: CC) yielded only slightly poorer performance in the same tests. None of the subsets gave better performance than the consolidated data set with either centrality measure – in fact, the MI IntAct reliable data set performed close to random as did the BioGrid and Rual and Stelzl subsets.

#### Analysis of reliable subsets of the full PIN

The fact that the full network has proven to be the best data source for drug target prediction over all other subsets (except the small MI-IntAct > 0.6 for degree) seemed counter-intuitive since we expected that some of these would contain more reliable data. We had reasoned that removing “unreliable” interactions might decrease the degree (connectivity) for some non-drug targets that had been artificially inflated and therefore reduce noise in the predictor due to false positives.

To test this reasoning, we evaluated the average change in the degree of a protein when losing edges from the full PIN to a “reliable” subset. Table 6 shows that drug targets in the NP data set have lost 5.2 edges on average compared to their degree in the full PIN, while non-drug targets only lose 2.7, which is a significant change (Wilcoxon p-value < 0.05). The same occurs when going from the full PIN to the B subset, indicating that drug targets lose more edges than non-drug targets, which is the opposite to what we expected if degree were a predictor of drug target status *and* our methods were actually able to extract more reliable data. Therefore, we reason that at least one of these two hypotheses is incorrect (see Discussion).

**Table 6:** Average change in degree for drug targets and non-drug targets after removing lower-confidence interactions

**Data sets**	**Avg degree change (drug targets)**	**Avg degree change (non-drug targets)**	**Wilcoxon p-value**
Full to non- predicted	−5.2	−2.7	3.7e-34
Full to B	−6.2	−4.9	9.3e-13
Full to LTP	−10.3	−9.7	0.5

After generating the true-binary (B) network, drug targets lose 6.2 edges on average compared to their degree in the full PIN. At the same time, non-drug targets lose 4.9 edges. This difference is statistically significant (p-value = 9.3e-13) for the two first cases, therefore we conclude that removal of lower-confidence data preferentially decreases the degree of drug targets rather than non-drug targets.

### Data Representation Analysis

Up to this point, a spoke-model has been used to represent n-ary data in the full network. We considered the effect of using a matrix-model instead to represent the same data. In this case, the average degree of the full human PIN is higher (42.86 for matrix versus 14.16 for spoke) (see Additional file 3: Table S2). Nevertheless, drug targets alone still have an average degree that is higher than other nodes in this network (61.69 for just drug targets). The relationship between drug targets and high-degree and high-centrality bins is still valid as well. However, the trend towards drug targets in higher degree bins is disrupted by a central spike and is far less clear (see Additional file 2: Figure S5). Table 7 shows that the degree distribution from the matrix representation of the PIN is (marginally) worse than from the spoke representation, probably due to the introduction of a high number of false positives in the matrix case. The three predictors maintain their order of effectiveness: BC followed closely by degree and then CC.

**Table 7:** Drug target predictive power of degree and centralities for spoke and matrix representation of protein complexes

**Network**	**AUC - Degree**	**AUC – BC**	**AUC – CC**
Full PIN, spoke	0.6139	0.6294	0.5795
Full PIN, matrix	0.5965	0.6264	0.5740

A matrix model of the full PIN has a slightly inferior performance to its spoke counterpart for all predictors under consideration.

### Observations on the integration of interaction and pathway data

Pathways have been traditionally used in drug discovery in the context of studying proteins upstream and downstream of a target in a pathway. Several studies [44] have emphasized the importance of enriching pathway data with interaction data due to the small overlap between these two data sources: There are, on average, 10 proteins with no interaction data per pathway in the KEGG database [45] and 15% of the proteins in pathways have no interaction data, including remarkable cases such as “olfactory transduction”, which, to this date, contains 349 proteins without interaction information. Besides that, drug target counts suggest that pathway data might be a good predictive feature alternative to interaction data. For example, there are only 225 drug targets that have no corresponding pathway in KEGG. Only 18 KEGG pathways contain no drug targets, and 81 out of 229 KEGG pathways are significantly enriched in drug targets (hypergeometric score < 0.05). For example, the TCA cycle contains 23 drug targets out of 30 proteins, and the average percentage of drug targets in a KEGG pathway for the human PIN is 23.8%.

One could imagine employing a simple network analysis using pathways; the number of pathways that a protein is involved in could be counted as a “pathway-centrality” and assessed for its relationship with drug target status. However, pathways from multiple databases are not easily consolidated making it difficult to determine how many distinct pathways a protein is involved in. Pathway databases are highly inconsistent both in terms of the biological entities and reactions [46-48] described for the same pathway. The boundaries of a pathway can be subjective such that different start and end points may be chosen and reactions may be divided into separate pathways [47]. Further, pathway databases may differ in the number of intermediate steps [48] and some databases combine pathway variants in one pathway while others generate separate pathway records for each variant [49]. Finally, pathway definitions or ontologies may differ or be completely absent [46]. The BioCyc database [50] defines a metabolic pathway, as part of a single biological process in a single organism, regulated as a unit, and that is evolutionary conserved with boundaries defined as stable substrates (not intermediates) with high-degree, typically branching points [46]. It has been reported that, as a consequence of a different ontology, KEGG pathways may be on average 4.2 times larger than BioCyc pathways [46]. It has also been reported that reasons for this inconsistency must be comparison or data integration problems such as different identifiers for the same entity, which should be resolved before an integration effort [47].

As a consequence we are unable to perform our analysis on a consolidated data set (analogous to the above analysis on a consolidated interaction data set). Instead, we had to resort to three separate pathway-centrality analyses on each of three different databases keeping in mind that results might not be comparable between databases. Pathway records between databases may be redundant and overlapping making results difficult to interpret.

We first compared the distribution of drug targets and non-drug targets in three different pathway databases: PID, Reactome and KEGG. Table 8 shows that KEGG contains the greatest number of drug targets (72% of all UniProt drug targets), followed by Reactome and PID. Proteins from UniProt that have pathway data are enriched for drug targets. Proteins in UniProt that do not have pathways are not enriched for drug targets.

**Table 8:** Drug target distribution in different pathway databases

**Database**	**#drug target in database**	**#Non-drug target in database**	**% of proteins in database that are drug targets**	**% of all drug targets with pathway info**	**% of all non-drug targets with pathway info**
UniProt (all prots)	1953	113741	1.69	83.97	8.33
PID	394	1261	23.81	20.17	1.11
Reactome	1262	4215	23.04	64.62	3.71
KEGG	1414	7473	15.91	72.40	6.57

84% of all drug-targets under study have pathway information. KEGG includes the highest number of drug targets (72%), followed by Reactome (65%). The number of non-drug targets in each database is small compared to all non-drug targets in UniProt, suggesting that pathways might be enriched for drug targets. Only 1.69% of UniProt proteins are drug targets while they constitute 15.9%-23.8% of pathway databases. This observation is confirmed by comparing the percentages of drug targets and non-drug targets in UniProt to those per database: KEGG, for example, contains 72.4% of all drug targets but only 6.6% of all non-drug targets found in UniProt.

If we hypothesize that proteins present in many pathways might be important for the cell and, therefore, disease and treatment processes, then counting the number of pathways per protein (“pathway centrality”) might be a useful feature for drug-target status prediction. This method can be understood as a kind of knowledge-based betweenness centrality, where shortest paths are replaced by actual information on known pathways. The distribution of the number of pathways per protein is, however, different for the different databases. Table 9 shows that drug targets have a higher average number of pathways per protein than non-drug targets, and PID has the highest averages followed by KEGG. This, as we said, might be related to the definition of a pathway.

**Table 9:** Differences in number of pathways and AUC of pathway centrality in three different pathway databases

**Database**	**Avg # pathways per drug target**	**Avg # pathways per non-drug target**	**Max # pathways per drug target**	**Max # pathways per non-drug target**	**AUC – Number of pathways for proteins in one pathway or more**	**AUC – Number of pathways for proteins in zero pathways or more**
PID	4.13	2.32	44	30	0.59	0.60
Reactome	1.85	1.71	17	23	0.53	0.81
KEGG	3.99	2.74	51	51	0.62	0.83

Drug targets are, on average, crossed by more pathways than non-drug targets. However, these values are relative to each pathway database.

KEGG allows the best performance for pathway centrality when using only the data in its database, while Reactome performs poorly. However, including the UniProt proteins not present in each database as part of the analysis, leads to an increase in the performance, and having both KEGG and Reactome as data sources, and the pathway centrality as predictor, can be considered as the best prediction platform investigated here.

Drug targets are over-represented in all pathway centrality bins for all three databases under analysis (see Additional file 2: Figure S7). In order to compute the predictive power of the number of pathways per drug target, the ROC curves were generated. Table 9 summarizes the AUC of pathways per protein for the three databases using two different UniProt data subsets. The fifth column shows the AUC when only the proteins reported in that database are used (i.e., proteins involved in at least one pathway). We observe that KEGG is the best dataset while Reactome performs close to randomness. The sixth column shows the result of including all UniProt proteins in the analysis, i.e., all UniProt proteins with no pathway will have a value of zero. In this case, two databases are good drug target predictors, especially KEGG with 0.83. This simple pathway metric outperforms degree and centrality of interaction networks under any studied reliability and representation condition. However, this increase in performance is due to the fact that the majority of proteins in UniProt do not have pathway information.

### Disease Analysis

The previous results motivated us to perform three additional analyses examining the relationship between drug targets and disease.

First, we surveyed the distance between drug targets and known disease proteins (Methods). Table 10 shows the distribution of shortest path lengths between the drug targets and the nearest disease protein. The full PIN contains 2062 disease proteins and 1227 drug targets. The full PIN contains only 436 drug targets which, at the same time, are disease proteins, validating the idea that drug targets are not necessarily disease proteins. For 619 different drug targets, the shortest path to a disease protein is 1, meaning that they interact. The shortest path is not necessarily the path that the drug follows to treat the disease, but we argue here that order-zero and order-one drugs give a rough idea of the number of “etiological” drugs while the rest might be considered as “palliative”. Five drug targets are disconnected from any disease target, which might indicate missing interaction information.

**Table 10:** Distribution of shortest path lengths from drug targets to the nearest disease protein

**Distance**	**Description**	**Full PIN**	**BioGRID**	**Rual-Stelzl**	**Rual-only**
0	Drug targets = DPs	436	319	71	25
1	Drug targets interact with DPs	619	246	47	10
2	Drug targets and DPs have a common interactor	154	163	77	25
3	3-step paths	12	16	20	11
4	4-step paths	1	1	1	2
5	5-step paths	-	1	-	-
Inf	Drug targets disconnected from DPs	5	5	5	1

The full PIN contains only 436 drug targets which, at the same time, are DPs. 619 different drug targets have a shortest path of “1” to the nearest DP (they interact), while 154 have a shortest path of “2” and 5 drug targets are disconnected from any disease target, probably due to missing interaction information. Smaller subsets show that, in general, drug targets do not get farther from disease proteins after data sub-setting, and, as a rule of thumb, there will always be a disease protein at least 4 steps away from any drug target. However, the proportion of drug targets in disconnected components is higher for subsets than it is for the full PIN.

Smaller subsets in Table 10 show that, in general, drug targets do not get further from disease proteins after data sub-setting, and, as a rule of thumb, there will always be a disease protein at least 4 steps away from a drug target. However, the proportion of drug targets disconnected from disease proteins is higher for subsets than it is for the full PIN.

Second, we hypothesized that degree might actually constitute a better predictor when applied to a subset of diseases. For example, high degree has already been noted as a feature of drug targets related to cancer [18]. We performed over-representation analysis of GADB disease categories in the consolidated PIN (see Methods) [51,52] and found that terms related to cancer and aging were over-represented for the highest-degree proteins while terms for most other diseases were not present (see Additional file 3: Table S3). Therefore, we reassessed degree as a predictor for drug targets related to cancer alone versus non-cancer related drug targets. ROC analysis of degree and centrality as predictors of only cancer drug targets (303 cancer drug targets out of 1227 drug targets), revealed that both performed better than when used to predict all drug targets in general. For example, Table 11 shows that the BC metric has an AUC of 0.6617 when applied solely to prediction of cancer drug targets which confirms that degree and centrality are better predictors for cancer drug targets only. Degree and BC have a more modest performance for non-cancer drug targets. This confirms that cancer proteins have a distinct behaviour with respect to drug target prediction and could be treated separately. We speculate that, in general, disease type may be an important feature that could be used in combination with degree and centrality as predictors of drug targets. Further study is warranted.

**Table 11:** Predictive power of degree and centralities for cancer and non-cancer drug targets

**Drug targets**	**# Proteins**	**AUC - Degree**	**AUC - BC**	**AUC – CC**
Cancer drug targets	303	0.6482	0.6617	0.6193
Non-cancer drug targets	924	0.5976	0.6133	0.5627

Third and finally, we hypothesized that highly central proteins could lead to more side-effects and, therefore, their drugs would be withdrawn from the market. Indeed, we found that the average BC of the subset of drug targets for withdrawn drugs is 54084.4 with a maximum of 1501217, which indicates that withdrawn drug targets have, on average, higher centralities than all drug targets and, of course, than the average of centralities in the full PIN (Wilcoxon p-value = 9.5e-6). In contrast, non-withdrawn drug targets have an average BC of 21411.7 and a maximum of 6930614, which is similar to the average and maximum values of the full PIN (Wilcoxon p-value = 0.8). These observations argue that high centrality should not be used as a predictor and may, in fact, be indicative of drugs that are more likely to be withdrawn.

## Discussion

Using the full PIN (iRefIndex consolidated data set) gives better prediction results than using presumably more reliable subsets such as the true binary interactions, low lpr score, non-predicted interactions, high IntAct MI score and high PSICQUIC MI score, and significantly better than using arbitrary subsets such as one given database or study. This could be taken as an argument in favour of the importance of interaction data integration in drug target prediction studies.

The poor performance of more reliable data sets compared to the full PIN might be due to one of two reasons. Either the subsets we are calling “reliable” are not as reliable as we think they are (and better definitions of reliability are needed) or, if we assume that our data is truly reliable, it is possible that the correlation of drug targets with degree and centralities is partially due to the inclusion of unreliable interactions. Both hypotheses demand further study. We would argue that our results also point out the need for more reliable interaction data and/or methods to filter for such data.

Representation issues seem to be less important for drug target prediction. Spoke models perform slightly better than matrix models, although the difference is not high. This might be due to the fact that most drug targets are present in binary interactions and not affected by complex representation.

Pathways are enriched in drug targets, only partially overlap with interaction data and the number of pathways that crosses a given protein seems to be a good drug target predictor. This could be interpreted as a need to integrate pathway data to the drug target prediction analysis, but also can be the reflection of the fact that the drug discovery process has been mainly pathway-oriented. However, as a consequence of the high inconsistency between pathway databases, an integration effort is required for pathways, similar to the iRefIndex for interaction data. There are integration efforts such as ConsensusPathDB [53], which highlights similar reactions and leaves to the user the decision of considering if they are identical or not. We believe that distributing pathways into pairwise interactions (such as pioneered by Reactome) and consolidating these interactions using a methodology such as iRefIndex's ssh keys (ROGs) [19], might be a better procedure to allow pathway integration and integration to PINs.

Our analysis can be improved in several ways. First, we are aware that degree and centralities might not be the best drug target prediction metrics and the analysis could be enriched by using better metrics and using an ensemble of features [14,15]. However, for the three tested metrics, all the conclusions regarding importance of data integration, negative effect of selecting reliable subsets and neutral effect of data representation, were consistent among the three metrics, making us expect a similar behaviour from more sophisticated prediction metrics. Second, as stated above, degree and centralities seem to be better predictors for cancer, therefore studies related to each type of disease would be recommended. And third, the fact that centralities are better predictors of withdrawn drugs also deserves a deeper analysis.

Even though our purpose was not to examine the predictive power of degrees and centralities compared to other metrics, but only their variation due to a different data source, our analysis has given us an important insight on how these metrics work and their limitations. Data type distinction, over-representation analysis and ROC curves have given us a deeper understanding of the reasons for and against using degree and centralities as drug target features and can be a methodology to use in the assessment of new prediction metrics.

## Conclusions

These initial results suggest that data integration is an important consideration when examining potential features for drug target prediction. Using more reliable data sets as defined here has little effect although other measures of confidence may have different results. The representation issues under analysis (n-ary data, matrix representation) do not have a significant effect on the predictive power of degree and centralities. This work will be of use to future studies that incorporate network data as a feature of drug target predictors.

## Methods

All analyses were performed using R and some of its packages: Â«iRefRÂ» for manipulation of the protein interaction database iRefIndex; Â«igraphÂ» for network analysis; Â«momentsÂ» for computation of statistical moments; Â«limmaÂ» for generating Venn diagrams; “plotrix” for multiple histograms; and Â«org.Hs.eg.dbÂ» for conversion between gene IDs and GO and pathway information. R code to generate all networks, tables and plots is provided as Additional file 1.

### Construction of Networks

Networks were constructed and analyzed using the iRefR package [33].

### Construction of the full PIN

The iRefIndex human MITAB file v.8.0 contains 355104 unique records, of which 309726 correspond to human-human interactions. Using a canonical representation of the proteins and including data with all levels of confidence, two protein interaction networks can be obtained: Using a spoke model to represent complexes, the PIN (full PIN, spoke) contains 16078 nodes and 113834 edges. Using a matrix model to represent complexes, the PIN (full PIN, matrix) contains 16078 nodes and 344576 edges. Even though drug targets may be dependent on post-translational modifications and cellular micro-environments [54], we have focused on the canonical representations of proteins, as described in the iRefIndex [33,38].

### Construction of the Drug target List

There are several drug target databases, such as DrugBank [55], SuperTarget [56], TTD [57], PharmGKB [58] and others. For the purposes of this paper, we have chosen DrugBank, but the reader can use the included R code (Additional file 1) in order to reproduce these analyses with any other drug target database.

A MITAB representation of the DrugBank database was retrieved, where the drug is described in the first field of the interaction and the drug target in the second field. The DrugBank MITAB table from September 2011 contained 40274 records, 19500 of which correspond to proteins. 14851 of those protein records were found in iRefIndex and only 12632 of these are human proteins.

DrugBank includes an “experimental” category of drugs, defined as “Drug has been shown experimentally to bind specific proteins in mammals, bacteria, viruses, fungi, or parasites. An experimental drug is not necessarily being formally investigated” [59]. Some studies remove this type of drug from the analysis due to the fact that they haven't proven efficacy against diseases. We follow the same line of thought and found 7032 records containing experimental drugs, from which 5011 correspond to human drug targets, and 7819 records containing non-experimental drugs, from which 7621 correspond to human proteins. As a result, 7621 records out of 40274 are useful for the purposes of this study.

These 7621 DrugBank records contain 1266 distinct protein drug targets. 1227 out of these 1266 drug targets belong to human-human protein interactions; therefore, this is the final number of drug targets that was studied.

It is important to highlight that the subset of non-iRefIndex drug targets contains 1592 proteins, which means that interaction data is missing (drug targets don't have a single known protein interaction in iRefIndex's databases) for more than half of the DrugBank human drug targets.

### Construction of drug target and non-drug target subnetworks

Drug target and non-drug target subnetworks were constructed using the “igraph” R package [60] and the spoke version of the full PIN. The drug target subnetwork contains 1227 nodes and 1038 edges (drug target-drug target interactions). The non-drug target subnetwork contains 14851 nodes and 94026 edges (interactions between non-drug-targets).

### Generating interaction-type sub-networks

The iRefIndex classifies interaction data according to three interaction types: Binary interaction records, n-ary interaction records (N) and polymers (not studied here). The S subset (spoke-represented n-ary data) corresponds to data that is represented as binary but is possibly just a representation of n-ary data. The S subset was detected using a simple algorithm: binary interaction records annotated by the same database from the same paper which were generated according to an experimental method that is known to generate n-ary data were grouped together into one S-type record [33]. Graphs containing just binary, n-ary or S-type data, were generated using the iRefR package [33]; their sizes are summarized in (Additional file 3: Table S4).

### Generating high-confidence subnetworks

Using the iRefR package [33], four main reliability criteria were considered: excluding predicted interactions from the interaction network, excluding interactions from high-throughput studies by using an lpr score smaller than 22, including only interactions with a high MI score – IntAct (> 0.6) or a high MI score – PSICQUIC (> 0.7).

The MI score tables were generated using a python script that submits iRefIndex interaction records, one at the time, to the scoring servers [41] and receives and consolidates these scores in an iRefIndex MITAB format. The algorithm to compute the scores is explained in [42]. The difference between both methods is that the first one includes information from IntAct only while the PSICQUIC version includes interaction data from all PSICQUIC servers (APID, ChEMBL, BioGrid, IntAct, DIP, InnateDB, MPIDB, iRefIndex, MatrixDB, MINT, Interoporc, Reactome, Reactome –FIs, STRING, BIND, DrugBank, I2D, I2D –IMEx, InnateDB –IMEx, and MolCon).

In order to select the cut-off values for each score type, 9 networks were generated for each score and the ROC test was applied to each of them. Values of 0.6 (for MI score - Intact) and 0.7 (for the MI score - PSICQUIC) had the highest AUC values and were chosen as cut-offs in this study. Additional file 3: Tables S5 and S6 show the sizes of all these networks.

### Prediction methods

Degree: Number of edges for a node or number of interactions for a protein. For computations, the igraph R package was used [60].

Centrality: Node centrality is a measure of the relative importance of a node within a graph. In our case, the relative importance of a protein inside a PIN. There are various ways to calculate centrality; in this study we used the most common measures called “betweenness” and “closeness” centralities. The Betweenness Centrality is a measure of the number of shortest paths that cross a given node. A node that is found in many shortest paths will have a higher betweenness centrality than a node that is not. The Closeness Centrality is a measure of the mean shortest distance between one node (protein) and all the others that it can reach, which is a measure of how long it will take information to spread from that node to the rest of network. For computations, the “igraph” R package [60] was used. igraph includes functions to calculate both centrality measures plus other less common types of centrality.

GO enrichment: When examining disconnected components, we considered “enriched” as the most common GO terms associated with a given subset of proteins. The “org.Hs.eg.db” R package [61] was used to convert gene IDs to GO terms. A routine to count the number of GO terms is included in the supplementary R code.

Pathway Centrality: We defined pathway centrality of a protein as the number of known biological pathways that cross that protein. For computations, the Â«org.Hs.eg.dbÂ» R package [61] was used to map gene IDs to pathways.

### Estimation of predictive power

The Receiver Operating Characteristic (ROC) or ROC curve is a plot of the True Positive Rate (TPR) versus the False Positive Rate (FPR), calculated as follows:

(1) $$FPR=FP/\left(FP+TN\right)$$

(2) $$TPR=TP/\left(TP+FN\right)$$

where FP = False Positives, TN = True Negatives, TP = True Positives, and FN = False Negatives.

The area under this curve (AUC) is interpreted as the probability that the classifier can rank a positive example better than a negative one, and here is calculated using a simple trapezoidal rule. We note that alternatives to the ROC method could be considered [62,63] as measures of performance.

### DAVID disease over-representation analysis

Proteins were grouped in bins of 700 proteins, from higher to lower degree, where bin 1 contained proteins with the highest degree. Each bin was submitted to DAVID [51,52] and results of over-represented GADB disease categories were summarized in the (Additional file 3: Table S3).

## Competing interests

The authors declare that they have no competing interests.

## Authors' contributions

AM performed all analyses in this paper and wrote all code for those analyses. IMD supervised the project. AM and IMD wrote this paper. All authors read and approved the final manuscript.

## Supplementary Material

Additional file 1 **This is a plain text file that contains R code to reproduce all R analyses in the paper.** See http://www.r-project.org/. Click here for file

Additional file 2 Supplementary Figures 1–8. Click here for file

Additional file 3 Supplementary Tables 1–6. Click here for file
