# Supplementary material for: Effects of protein interaction data integration, representation and reliability on the use of network properties for drug target prediction
Source: BMC Bioinformatics. 2012 Nov 12;13:294. doi: 10.1186/1471-2105-13-294 (PMC3534413; doi:10.1186/1471-2105-13-294)
Supplement: Additional file 2 — Supplementary Figures 1–8. [file 1471-2105-13-294-S2.pdf]

Additional file 2 – Supplementary Figures

Supplementary Figure 1 – DT degree plot.
The plot highlights the line corresponding to the 95th percentile, showing that most DTs have lower degree.

Supplementary Figure 2 – Comparative histogram of DTs and non-DTs over a degree rank.

Supplementary Figure 3 - Over-representation of drug targets over a BC ranking of nodes in the full spoke PIN, Binary (B), N-ary (N), Spoke-represented (S), BioGRID and Rual+Stelzl data sets.
Proteins were grouped into bins according to their BC. The width of each bin represents the number of proteins in that bin while the height (-log of the p-value of the hypergeometric test) represents how over-represented drug targets are in that bin. Each bin contains at least 200 proteins. Over-represented bins (p-value < 0.05) are highlighted in red. Drug targets are over-represented in high-degree bins and some middle-degree bins for the full PIN (a) and the B data set (b), while this trend is largely lost in the N (c), S (d), BioGRID (e) and the Rual-Stelzl (f) data sets.

Supplementary Figure 4 – ROC curve for BC as DT predictor (full PIN and reliable subsets).
Plot of False Positive Rate versus True Positive Rate for a BC rank of the full PIN and five subsets considered as containing higher-confidence interactions: non-predicted interactions include all interactions except those coming from orthologous transfer; LTP includes interactions with an lpr score < 22; MI-IntAct includes interactions with MI-IntAct scores > 0.6; MI-psicquic includes interactions with MI-psicquic scores > 0.7; and B includes the true binary interactions (i.e., potential spoke-represented n-ary data is removed).

Supplementary Figure 5 - Over-representation of drug targets along a degree rank and a BC rank for the full PIN with a matrix representation of protein complexes.
Proteins were grouped into bins according to their degree (a) and their BC (b), for a matrix model of the full PIN. The width of each bin represents the number of proteins in that bin while the height (-log of the p-value of the hypergeometric test) represents how over-represented drug targets are in that bin. Each bin contains at least 200 proteins. Over-represented bins (p-value < 0.05) are highlighted in red. Drug targets are over-represented in high-degree bins and some middle-degree bins, just as in the spoke representation.

Supplementary Figure 6 – ROC curve for degree (spoke and matrix), and BC (matrix).
Plot of False Positive Rate versus True Positive Rate for a degree rank of the full PIN following a spoke and a matrix representation of protein complexes, and a BC rank of the full PIN following a matrix representation of protein complexes.

Supplementary Figure 7 - Over-representation of drug targets according to pathway centrality.
Proteins were grouped into bins according to their pathway centrality. The width of each bin represents

the number of proteins in that bin while the height (-log of the p-value of the hypergeometric test) represents how over-represented drug targets are in that bin. Each bin contains at least 200 proteins. Over-represented bins (p-value < 0.05) are highlighted in red. Drug targets are highly over-represented in all pathway centrality bins greater than zero and are not over-represented in proteins outside the pathway databases (pathway centrality = 0 – not shown). This observation is valid for all databases: PID (a), Reactome (b) and KEGG (c). A positive correlation occurs between increasing pathway centrality and over-representation only for the PID database.

Supplementary Figure 8 – ROC curve for pathway centrality over different pathway databases. Plot of False Positive Rate versus True Positive Rate for a pathway centrality rank of the Reactome, PID and KEGG pathway databases.

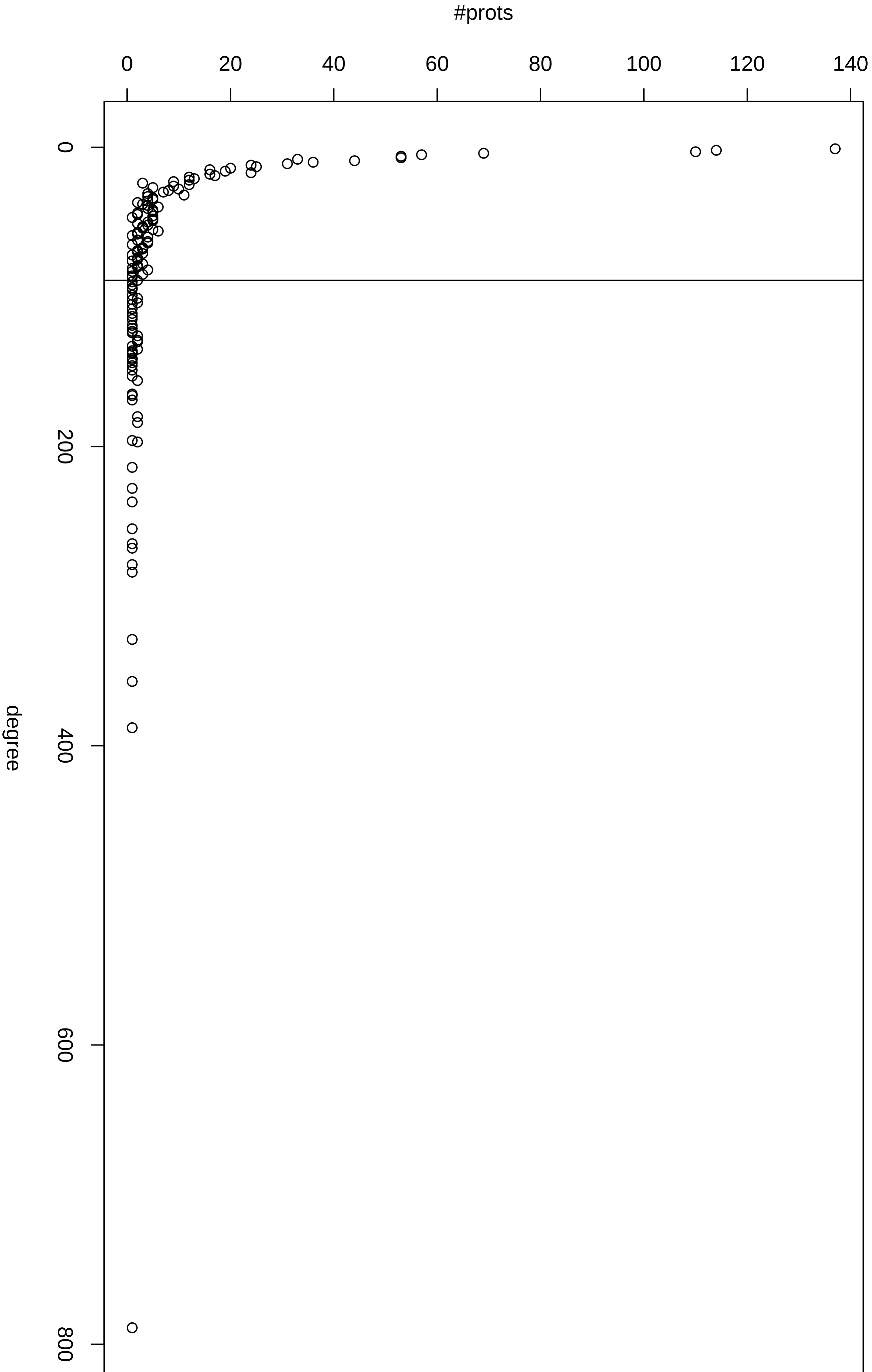

#prots
0
20
40
60
80
100
120
140
0
200
400
600
800
degree

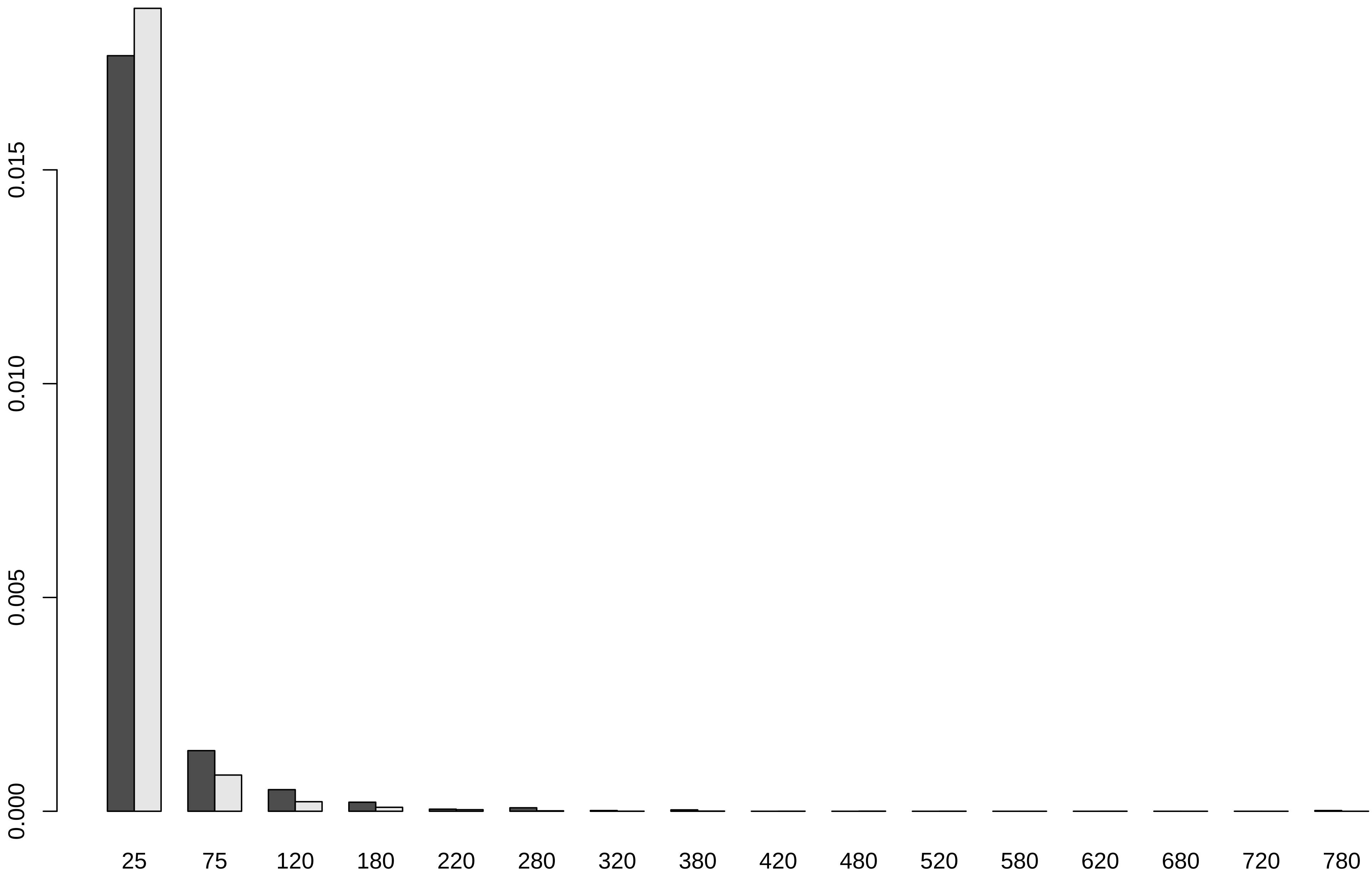

0.000
0.005
0.010
0.015
25
75
120
180
220
280
320
380
420
480
520
580
620
680
720
780

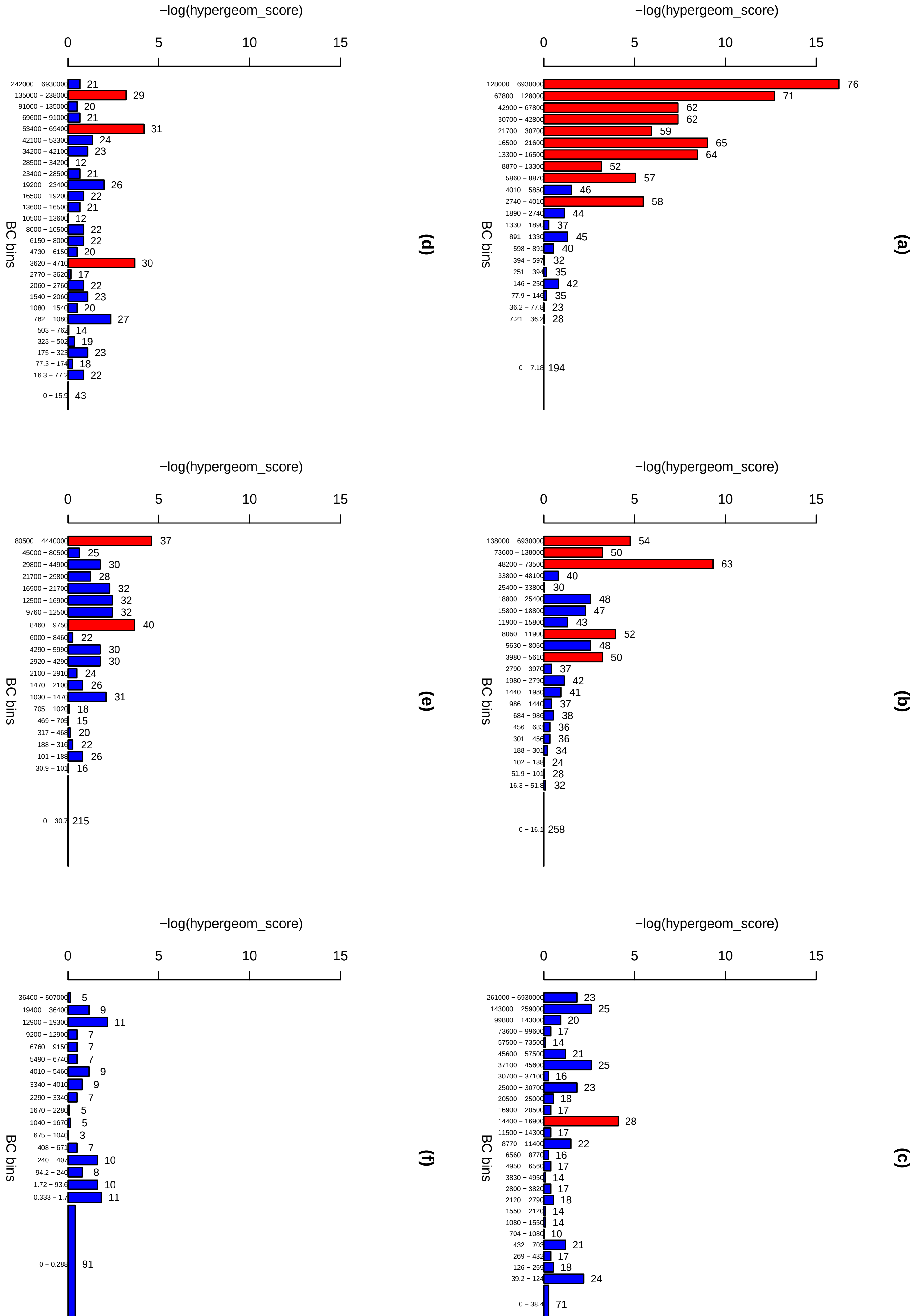

−log(hypergeom_score)
BC bins
(a)
(b)
(c)
(d)
(e)
(f)

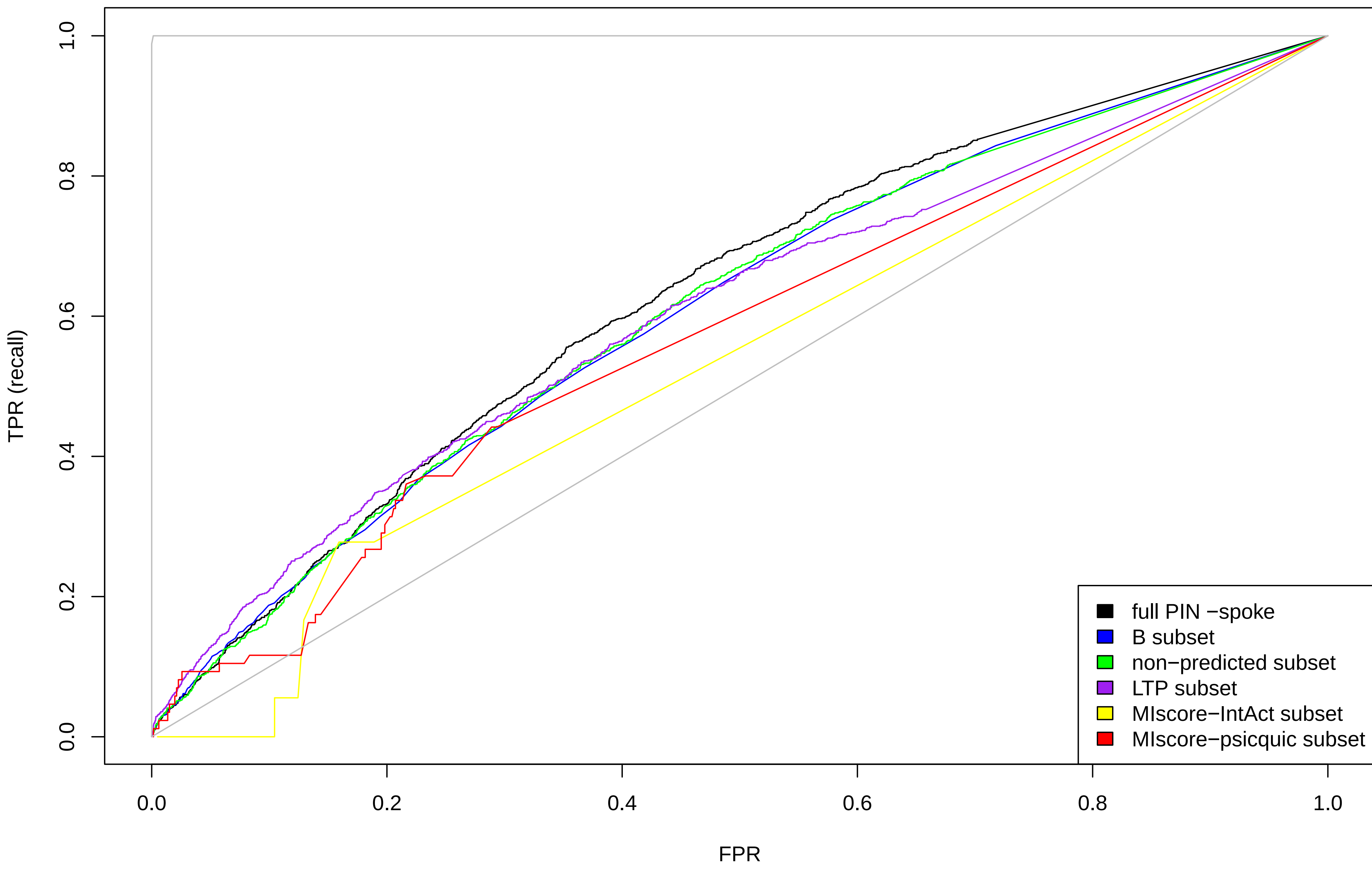
TPR (recall)
FPR
0.0
0.2
0.4
0.6
0.8
1.0
full PIN −spoke
B subset
non−predicted subset
LTP subset
MIscore−IntAct subset
MIscore−psicquic subset

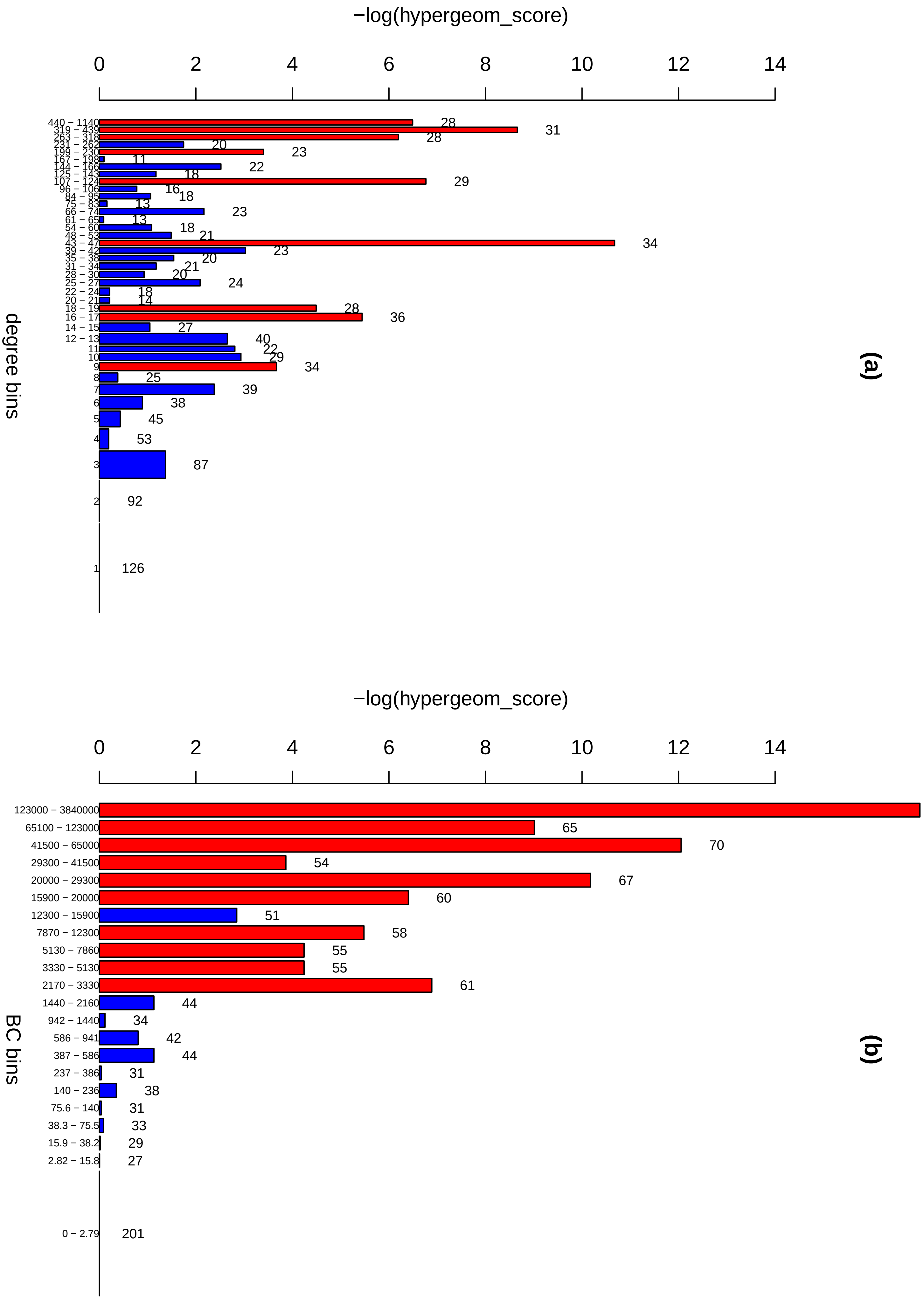
−log(hypergeom_score)
0
2
4
6
8
10
12
14
degree bins
440 − 1140
319 − 439
263 − 318
231 − 262
199 − 230
167 − 198
144 − 166
125 − 143
107 − 124
96 − 106
84 − 95
75 − 83
66 − 74
61 − 65
54 − 60
48 − 53
43 − 47
39 − 42
35 − 38
31 − 34
28 − 30
25 − 27
22 − 24
20 − 21
18 − 19
16 − 17
14 − 15
12 − 13
11
10
9
8
7
6
5
4
3
2
1
28
31
28
20
23
11
22
18
29
16
18
13
23
13
18
21
34
23
20
21
20
24
18
14
28
36
27
40
22
29
34
25
39
38
45
53
87
92
126
(a)
−log(hypergeom_score)
0
2
4
6
8
10
12
14
BC bins
123000 − 3840000
65100 − 123000
41500 − 65000
29300 − 41500
20000 − 29300
15900 − 20000
12300 − 15900
7870 − 12300
5130 − 7860
3330 − 5130
2170 − 3330
1440 − 2160
942 − 1440
586 − 941
387 − 586
237 − 386
140 − 236
75.6 − 140
38.3 − 75.5
15.9 − 38.2
2.82 − 15.8
0 − 2.79
65
70
54
67
60
51
58
55
55
61
44
34
42
44
31
38
31
33
29
27
201
(b)

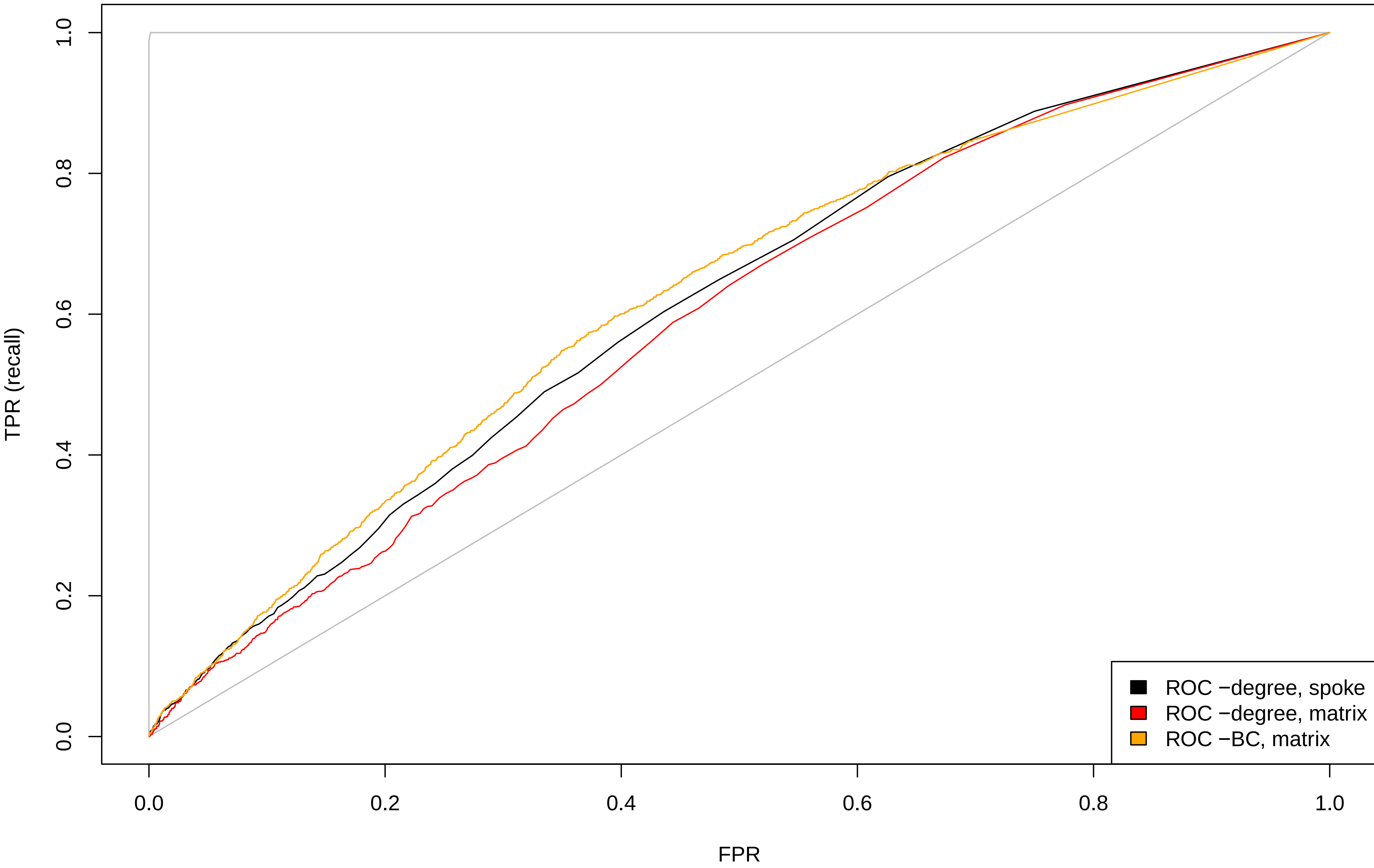

TPR (recall)
FPR
0.0
0.2
0.4
0.6
0.8
1.0
ROC −degree, spoke
ROC −degree, matrix
ROC −BC, matrix

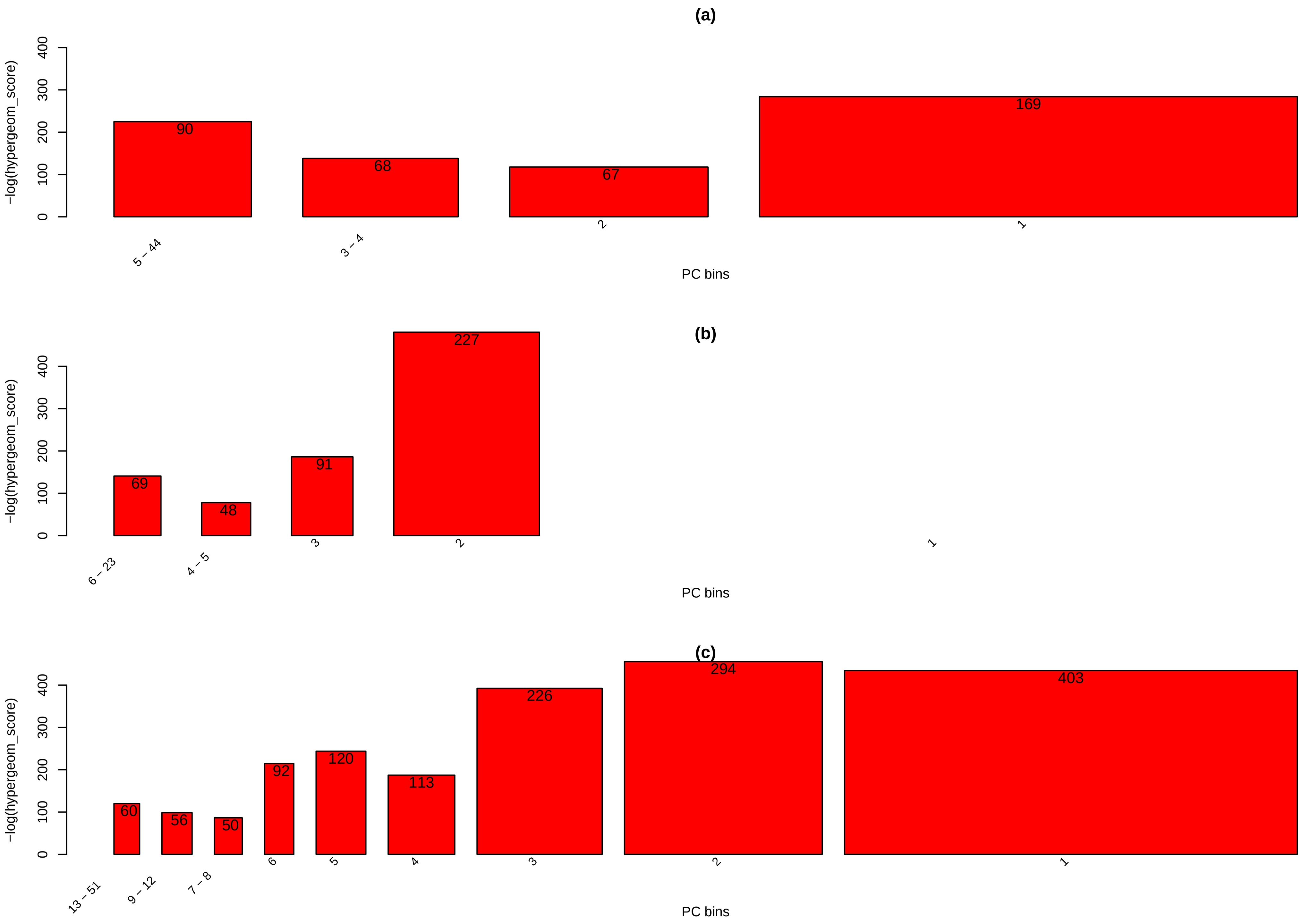
(a)
−log(hypergeom_score)
0
100
200
300
400
90
68
67
169
5 – 44
3 – 4
2
1
PC bins
(b)
−log(hypergeom_score)
0
100
200
300
400
69
48
91
227
6 – 23
4 – 5
3
2
1
PC bins
(c)
−log(hypergeom_score)
0
100
200
300
400
60
56
50
92
120
113
226
294
403
13 – 51
9 – 12
7 – 8
6
5
4
3
2
1
PC bins

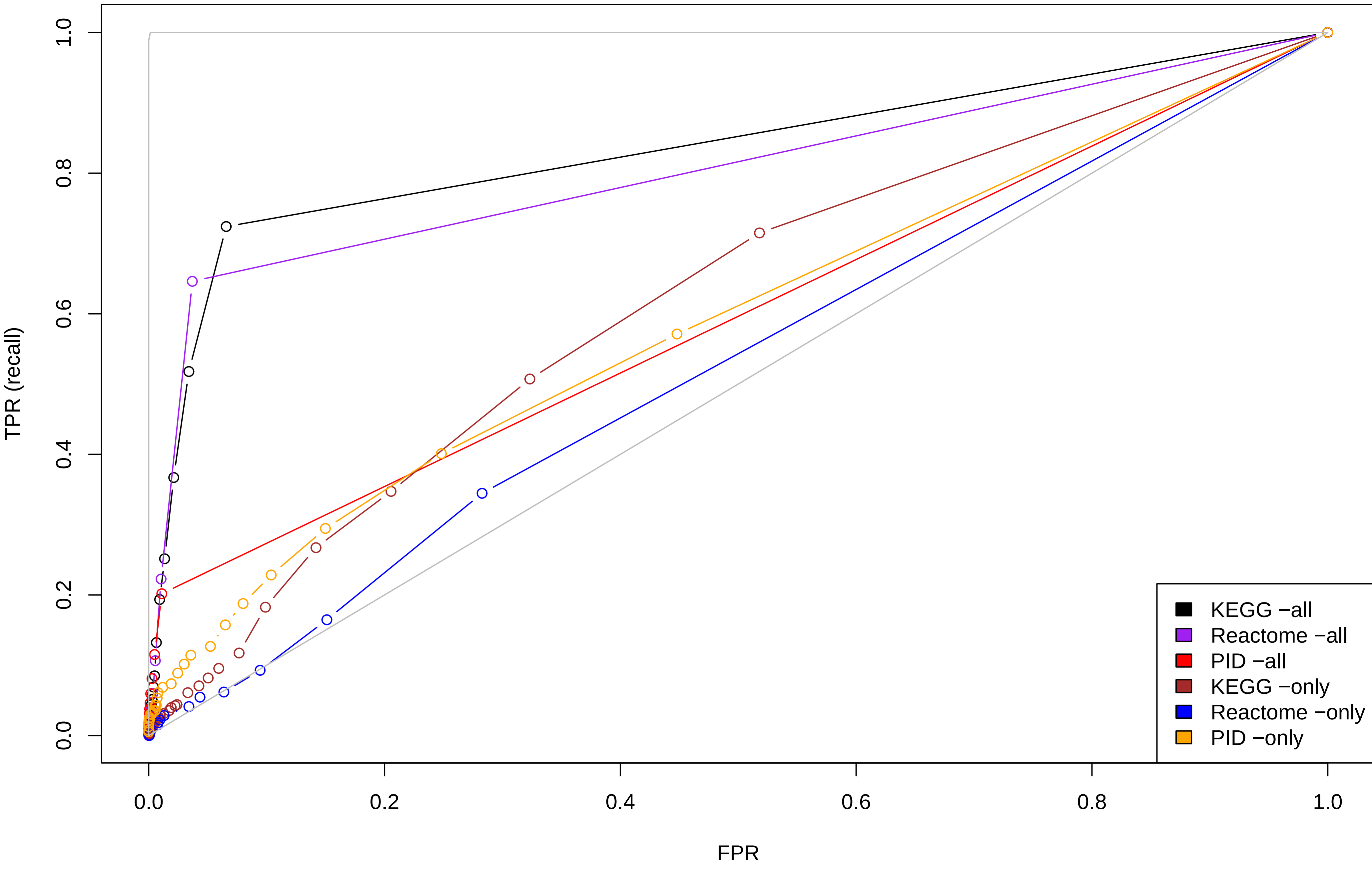

TPR (recall)
FPR
0.0
0.2
0.4
0.6
0.8
1.0
KEGG −all
Reactome −all
PID −all
KEGG −only
Reactome −only
PID −only
