# Supplementary material for: Effects of protein interaction data integration, representation and reliability on the use of network properties for drug target prediction
Source: BMC Bioinformatics. 2012 Nov 12;13:294. doi: 10.1186/1471-2105-13-294 (PMC3534413; doi:10.1186/1471-2105-13-294)
Supplement: Additional file 3 — Supplementary Tables 1–6. [file 1471-2105-13-294-S3.pdf]

Additional file 3 - Supplementary Tables

Supplementary Table 1 - Connected component distribution of the full spoke human PIN.

# Members in component	1	2	3	4	5	8	27	15754
# Components	23	88	20	3	2	2	1	1

There is one giant component and several smaller components in the full spoke human PIN. The components with one member only correspond to proteins that only have polymeric interactions in iRefIndex, and include metabolic enzymes such as F264, pore-forming ion channels such as CALHM1, structural components of the eye lens as CRGD, and collagen-alpha-1 proteins, involved in cell binding.

Supplementary Table 2 – Degree of all proteins and DTs after the spoke and matrix representation of protein complexes.

PIN	avg degree	sd	max degree
Full graph –spoke	14.16	29.01	789
DT in full graph –spoke	22.48	44.94	789
Full graph –matrix	42.86	93.49	1145
DT in full graph -matrix	61.69	115.51	889

Supplementary Table 3 – Over-representation analysis of GADB disease classes in high-degree bins.

Bin ID	Valid DAVID IDs (out of 700 in each bin)	GADB Disease Class	Number of Proteins	P-values < 0.1
Bin 1	622	CANCER	94	2.68E-13
		AGING	17	0.0009
		OTHER	60	0.0265
		NEUROLOGICAL	47	0.0356
		PHARMACOGENOMIC	28	0.0757
Bin 2	633	CANCER	75	2.53E-08
Bin 3	620	CANCER	57	0.0040
		AGING	11	0.0594
Bin 4	634	CANCER	55	0.0108
		UNKNOWN	20	0.0351
		VISION	16	0.0653
		OTHER	49	0.0872

		REPRODUCTION	19	0.0995
Bin 5	632	UNKNOWN	22	0.0152
		OTHER	55	0.0187
		CANCER	55	0.0258
		REPRODUCTION	22	0.0287
		HEMATOLOGICAL	14	0.0510
		RENAL	16	0.0623
		DEVELOPMENTAL	21	0.0663
		CARDIOVASCULAR	47	0.0665
		INFECTION	23	0.0986
Bin 6	634	CARDIOVASCULAR	50	0.0078
		IMMUNE	56	0.0115
		OTHER	52	0.0257
		UNKNOWN	19	0.0591
		INFECTION	23	0.0648
		NEUROLOGICAL	39	0.0730
Bin 7	637	CARDIOVASCULAR	53	0.0011
		IMMUNE	56	0.0087
		METABOLIC	57	0.0158
		UNKNOWN	20	0.0296
		VISION	16	0.0571
		CHEMDEPENDENCY	15	0.0611
		OTHER	49	0.0660
Bin 8	645	NEUROLOGICAL	46	0.0012
		PHARMACOGENOMIC	27	0.0137
		PSYCH	40	0.0174
		NORMALVARIATION	23	0.0426
		INFECTION	23	0.0484
		UNKNOWN	18	0.0791
		CARDIOVASCULAR	43	0.0885
		OTHER	47	0.0986
Bin 9	639	-	-	-
Bin 10	635	-	-	-
Bin 11	645	REPRODUCTION	20	0.0180
		UNKNOWN	18	0.0377
		IMMUNE	47	0.0490
		NORMALVARIATION	21	0.0521
		INFECTION	21	0.0585

		PHARMACOGENOMIC	22	0.0772
Bin 12	644	METABOLIC	48	0.0511
Bin 13	622	OTHER	42	0.0134
Bin 14	611	CHEMDEPENDENCY	14	0.0324
		RENAL	13	0.0773
Bin 15	617	NORMALVARIATION	21	0.0044
Bin 16	610	-	-	-
Bin 17	611	-	-	-
Bin 18	590	NORMALVARIATION	18	0.0045
		DEVELOPMENTAL	15	0.0149
Bin 19	573	HEMATOLOGICAL	9	0.0290
		NEUROLOGICAL	23	0.0293
		NORMALVARIATION	13	0.0719
		IMMUNE	27	0.0955
Bin 20	605	-	-	-

SupplementaryTable 4 - Size of interation-type networks.

Network	Description	Network Size
B	True Binary Data, spoke	14408 nodes, 75207 edges
N	N-ary Data, spoke	6203 nodes, 22847 edges
S	Spoke-represented complexes	7120 nodes, 26957 edges
Full	full graph, spoke	16078 nodes, 113834 edges

Supplementary Table 5 - Size of high-confidence networks.

Network	Description	Network Size
non-predicted	Full PIN spoke, excluding predicted interactions (OPHID database)	14928 nodes, 88014 edges
LTP	Full PIN spoke, excluding interactions with lpr < 22	10591 nodes, 45848 edges
MI score, IntAct	Full PIN spoke, excluding interactions with MI score (IntAct) > 0.6	219 nodes, 153 edges
MI score, Psicquic	Full PIN spoke, excluding interactions with MI score (Psicquic) > 0.7	747 nodes, 666 edges

Supplementary Table 6 - Number of nodes and edges for the human PIN for 9 different cutoff values of each MI score.

MIscore	> 0.1	> 0.2	> 0.3	> 0.4	> 0.5	> 0.6	> 0.7	> 0.8	> 0.9
IntAct	4125 n<br>7850 e	4125 n<br>7850 e	4109 n<br>7817 e	2584 n<br>4052 e	628 n<br>484 e	219 n<br>153 e	51 n<br>33 e	6 n<br>3 e	0 n<br>0 e
psicquic	6293 n<br>13030 e	5512 n<br>9960 e	5093 n<br>8836 e	4706 n<br>7838 e	3169 n<br>4419 e	1598 n<br>1735 e	747 n<br>666 e	286 n<br>215 e	11 n<br>7 e
